# Detection of early neoplasia in Barrett’s esophagus using lectin-based near-infrared imaging: an ex vivo study on human tissue

**DOI:** 10.1055/s-0043-124080

**Published:** 2018-01-17

**Authors:** André A. Neves, Massimiliano Di Pietro, Maria O’Donovan, Dale J. Waterhouse, Sarah E. Bohndiek, Kevin M. Brindle, Rebecca C. Fitzgerald

**Affiliations:** 1 Cancer Research UK Cambridge Institute, Li Ka-Shing Centre, Cambridge, UK; 2 Medical Research Council, Cancer Unit, University of Cambridge, Cambridge, UK; 3 Department of Histopathology, Cambridge University Hospitals, Cambridge, UK; 4 Department of Physics, University of Cambridge, Cambridge, UK

## Abstract

**Background and study aims** Endoscopic surveillance for Barrett’s esophagus (BE) is limited by long procedure times and sampling error. Near-infrared (NIR) fluorescence imaging minimizes tissue autofluorescence and optical scattering. We assessed the feasibility of a topically applied NIR dye-labeled lectin for the detection of early neoplasia in BE in an ex vivo setting.

**Methods** Consecutive patients undergoing endoscopic mucosal resection (EMR) for BE-related early neoplasia were recruited. Freshly collected EMR specimens were sprayed at the bedside with fluorescent lectin and then imaged. Punch biopsies were collected from each EMR under NIR light guidance. We compared the fluorescence intensity from dysplastic and nondysplastic areas within EMRs and from punch biopsies with different histological grades.

**Results** 29 EMR specimens were included from 17 patients. A significantly lower fluorescence was found for dysplastic regions across whole EMR specimens ( *P* < 0.001). We found a 41 % reduction in the fluorescence of dysplastic compared to nondysplastic punch biopsies ( *P* < 0.001), with a sensitivity and specificity for dysplasia detection of 80 % and 82.9 %, respectively.

**Conclusion** Lectin-based NIR imaging can differentiate dysplastic from nondysplastic Barrett’s mucosa ex vivo.

## Introduction

The incidence of esophageal adenocarcinoma (EAC) has increased dramatically in the Western world over the last 30 years. Despite treatment advances this cancer is still associated with a 5-year survival of less than 15 %, due principally to late diagnosis 1 . EAC has a defined pathological sequence with a precursor condition known as Barrett’s esophagus (BE) and intermediate premalignant stages categorized as low grade dysplasia (LGD) and high grade dysplasia (HGD). Even though the risk of malignant transformation in BE is small, estimated at around 0.3 % per year, endoscopic surveillance of BE is recommended 2 as early diagnosis of EAC is associated with improved patient outcome 3 .

Endoscopic surveillance is generally performed according to the Seattle protocol, which entails 4 biopsies every 2 centimeters within the BE segment, but is affected by sampling error due to inconspicuous dysplasia and sampling error 2 . Moreover, this protocol is time-consuming, poorly adhered to in routine practice, and very intensive for patients 4 . To improve endoscopic detection of dysplasia, several imaging modalities have been tested; however, evidence is lacking to show that these techniques can increase diagnostic accuracy compared with standard high definition white light endoscopy 5 .

The combination of molecular imaging tools with endoscopy may provide novel routes for better diagnosis 6 . We have previously shown that alterations in esophageal glycosylation patterns are candidate biomarkers for detection of early neoplasia associated with BE, using fluorescently labeled lectins as imaging probes 7 . Near-infrared (NIR) molecular imaging minimizes tissue autofluorescence and, in the endoscopy field, has been used in combination with monoclonal antibodies or peptides in preclinical ex vivo and in vivo models of colon cancer 8 9 .

Here we describe the synthesis and characterization of a novel, topically applied lectin-based probe for the endoscopic detection of early BE-related neoplasia, based on the conjugation of a lectin, wheat germ agglutinin (WGA), to a commercially available and clinically applicable NIR fluorophore (IR800CW) 10 . We demonstrate that this labeled lectin can be used to detect dysplasia in a human ex vivo model of BE neoplasia using NIR imaging.

## Methods

### Study population

The study was approved by the Cambridgeshire-2 Research Ethics Committee (09/H0308/118).

Consecutive patients, referred for endoscopic management of Barrett’s-related neoplasia, were recruited prospectively between June 2014 and February 2016 at a single tertiary referral center ( Table1 ). Exclusion criteria were: evidence of stage > T1 on endoscopic ultrasound; previous upper gastrointestinal (GI) surgery (with the exception of Nissen fundoplication); coagulopathy or anticoagulant/antiplatelet therapy for high risk conditions; active or severe cardiopulmonary disease or liver disease; or special communication needs. Patients undergoing endoscopic mucosal resection (EMR) for BE-related early neoplasia were recruited.

**Table 1:** Clinical features of patients undergoing endoscopic mucosal resection (EMR) for Barrett’s esophagus-related early neoplasia, and fluorescence intensity of endoscopic mucosal resection specimens. 1

Patient no.	Age, years	Sex		Prague classification	Lesion size, cm	EMR	Highest pathology	Mean fluorescence intensity (SD)		Contrast, % 2	Dysplasia, % of area
								Nondysplastic	Dysplastic		
1	59	M	T2C59	C7M8	4	1	HGD	0.054 (0.02)	0.049 (0.013)	– 8.9 (4.1)	34.8
						2	HGD	0.105 (0.011)	0.089 (0.008)	– 16.3 (2.3)	32.0
						3	IMC	0.065 (0.047)	0.047 (0.025)	– 27.3 (24.5)	80.0
						4	HGD	0.061 (0.013)	0.049 (0.014)	– 20.1 (7.2)	60.7
						5	HGD	0.065 (0.004)	0.045 (0.015)	– 30.6 (10.6)	73.3
4	81	M	T2C65	C9M10	1	1	IMC	0.156 (0.027)	0.138 (0.016)	– 11.0 (2.3)	80.5
						2	IMC	0.244 (0.042)	0.216 (0.019)	– 11.4 (2.2)	10.8
5	58	M	T2C66	C3M6	1	1	IMC	0.243 (0.068)	0.205 (0.068)	– 15.7 (6.8)	72.0
						2	IMC	0.162 (0.023)	0.144 (0.035)	– 11.1 (3.1)	18.8
6	63	M	T2C71	C2M4	2	1	IMC	0.161 (0.040)	Focal/none 3	Not defined	0
						2	GM	0.160 (0.025)	Focal/none	Not defined	0
7	65	F	T2C72		1	1	HGD	0.106 (0.034)	Focal/none	Not defined	0
						2	HGD	0.171 (0.029)	0.166 (0.028)	– 2.7 (0.6)	20.5
8	80	F	T2C74	C0M1	1	1	HGD	0.269 (0.024)	0.252 (0.038)	– 6.1 (1.1)	19.1
						2	HGD	0.245 (0.045)	0.252 (0.010)	3.0 (0.6)	6.3
10	74	M	T2C76	C2M3		2	IMC	0.182 (0.047)	0.161 (0.041)	– 11.3 (4.1)	42.0
						3	HGD	0.168 (0.033)	Focal/none	Not defined	0
11	66		T2C77	GEJ		1	IMC	0.131 (0.013)	0.122 (0.008)	– 6.6 (0.8)	57.7
12	66		T2C78	C0M3		1	HGD	0.200 (0.031)	Focal/none	Not defined	0
						2	HGD	0.122 (0.020)	0.112 (0.007)	– 8.1 (1.4)	15.0
						3	LGD	0.175 (0.039)	Focal/none	Not defined	0
14	60		T2C80	C0M3		1	HGD/LGD	0.199 (0.049)	Focal/none	Not defined	0
15	52		T2C83	C2M4	1.5	1	HGD	0.148 (0.031)	Focal/none	Not defined	0
						2	HGD	0.186 (0.036)	Focal/none	Not defined	0
16	74		T2C84	C1M3		2	IMC	0.406 (0.087)	0.342 (0.072)	– 15.8 (4.8)	40.6
						3	IMC	Focal/none	0.358 (0.035)	Not defined	100
						4	IMC	0.313 (0.031)	0.299 (0.029)	– 4.3 (0.6)	29.2
						5	IMC	0.419 (0.089)	0.433 (0.065)	3.6 (0.9)	6.3
17	60		T2C87	C6M7		1	HGD	0.199 (0.039)	0.148 (0.025)	– 25.6 (6.6)	36.2

ID, identity; HGD, high grade dysplasia; IMC, intramucosal cancer; GM, gastric metaplasia; GEJ, gastroesophageal junction.

1 No data are available for the EMR specimens from patients 2, 3, 9 and 13 and for some specimens from patients 10, 11 and 16; this is because of failed specimen orientation or failed section mounting in the pathology lab.

2 Not defined indicates contrast not defined, because of absence of dysplasia.

3 Contrast = ( *D* – *ND* )/ *ND* × 100, where *D* is dysplastic, *ND* is non dysplastic

4 Focal/None indicates no dysplasia or focal dysplasia (i. e., very low levels, < 3 grid cell elements).

### Endoscopic procedure

Gastroscopies were carried out using FQ260Z endoscopes (Olympus, Tokyo, Japan). The neoplastic areas were assessed by a combination of white light endoscopy, narrow band imaging with magnification, and autofluorescence imaging for precise delineation of the lesion. EMR was carried out with a Duette multiband mucosectomy device (Cook Medical).

### Synthesis of wheat germ agglutinin (WGA) lectin – dye conjugates

WGA (L9640; Sigma, Missouri, US), was conjugated to IR800CW-NHS ester dye (Li-Cor, USA), and purified using modified versions of the methods described elsewhere 11 12 . The synthesis of WGA-IR800 conjugates was optimized so that an average of two dye molecules were incorporated per lectin.

### Staining protocol and biopsy collection

EMR specimens were collected from patients, washed with 5 mL of ice-cold phosphate-buffered saline (PBS), sprayed with 2 mL of WGA-IR800 (10 μg/mL) in PBS, incubated in the dark for 10 min at room temperature, and then washed again with the same buffer, prior to imaging using an intraoperative fluorescence imaging device (Fluobeam-800 CE Mark, Fluoptics, Grenoble, France). A maximum of two punch biopsies (2 mm diameter) were collected ex vivo from each EMR specimen under NIR fluorescence guidance. The combined staining, imaging, biopsy collection procedure was limited to a maximum time of 30 minutes, to ensure preservation of the specimens for subsequent histopathology. Following the procedure, EMR specimens and biopsies were immediately fixed in formalin.

### Pathological assessment

The EMR specimen paraffin block was cut at 2-mm intervals from the 12-o’clock to the 6-o’clock margin and single sections were mounted onto glass slides. Single sections were read every 1 mm, in a direction orthogonal to the original cut, by the pathologist.

Cases were reviewed by two pathologists with experience in upper GI neoplasia, including the study pathologist (M.O’D.) who has extensive experience in reporting Barrett’s esophageal neoplasia and a satisfactory level of interobserver agreement with external pathologists in previous studies 7 13 . Neoplasia was interpreted and reported in accordance with the Vienna classification 14 .

Excluded from the analysis were EMR specimens with no dysplasia or less than 3 dysplastic/neoplastic grid elements and EMR specimens that failed orientation.

### Statistics

Data are expressed as mean ± SD, unless stated otherwise. The two-tailed Wilcoxon matched-pairs signed rank test was used for pairwise comparison of dysplastic and nondysplastic areas of EMR specimens. The Mann–Whitney test was used for analysis of punch biopsy fluorescence. Correlation analysis was performed using the extra sum-of-squares *F* test. GraphPad Prism (V5, Sigma Software, Ashburton, UK) was used throughout in the analysis. *P* < 0.05 was considered statistically significant.

Because of the exploratory nature of this ex vivo imaging study and the absence of a previously acquired large dataset, a formal calculation of sample size was not possible. We anticipated that with an average of 50 data points per EMR specimen from the pathology grids ( Fig. 1c, d ), 20 EMR specimens would generate a sufficient number of fluorescence measurements to support statistical analysis.

**Figure:**
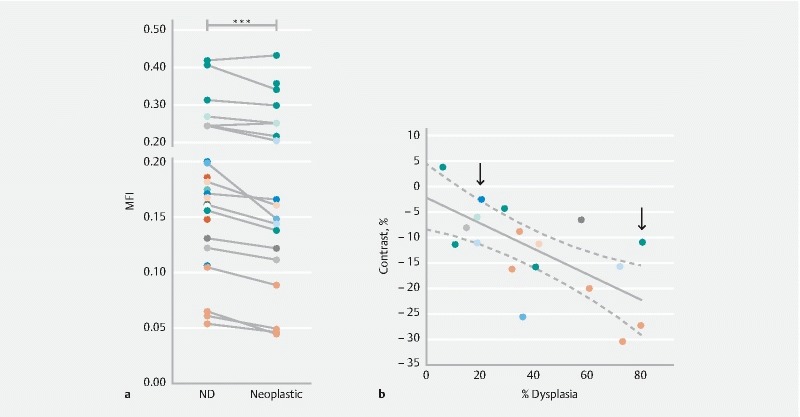


**Figure:**
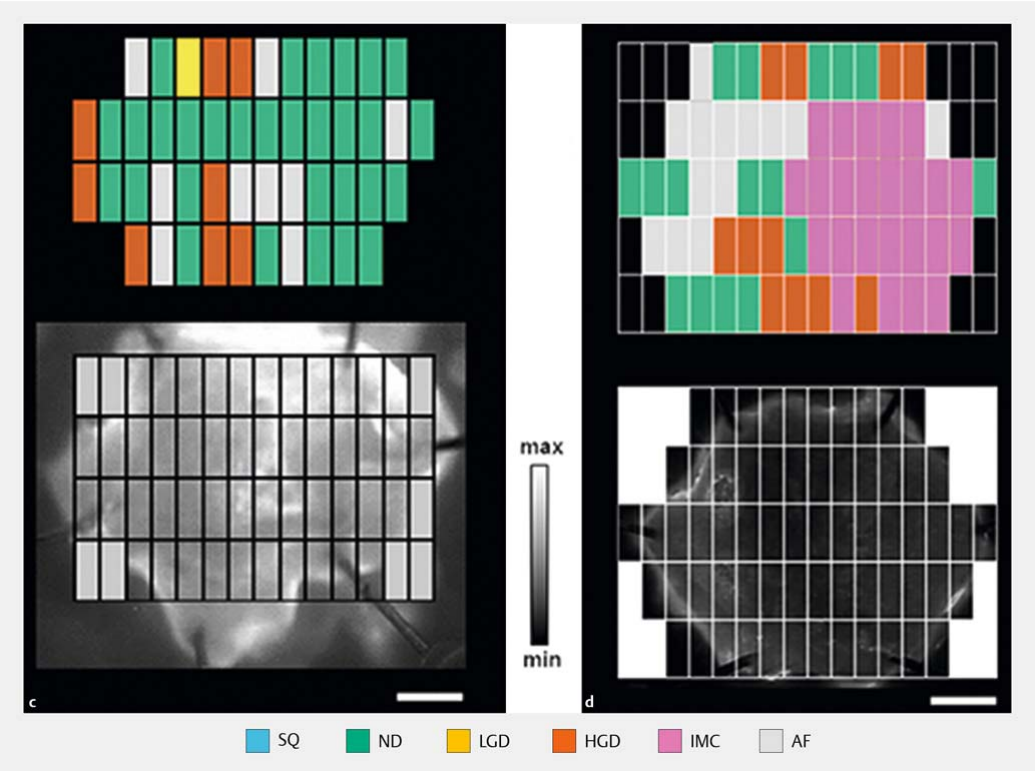


## Results

### Patient characteristics

A total of 21 patients were recruited into the study. After exclusion of 4 patients who presented with advanced lesions not amenable to EMR, 17 patients completed the endoscopy and ex vivo imaging protocol. The median age was 65 years (range 52 – 84), the average length of the Barrett’s segment was 4.2 cm (range 0.5 – 10 cm), and the median lesion size was 1 cm (range 1 – 4 cm). On average, 2 EMR specimens (range 1 – 5) were collected per patient, from which a median of 2 punch biopsies were collected per EMR (range 0 – 2).

### Association of dysplasia with fluorescence contrast in EMR

The fluorescence signal from EMR specimens, collected at the bedside and freshly stained with WGA-IR800, was analyzed in relation to pathology. Co-registration with the pathology grid allowed correlation of the histopathology for each specimen level and for each grid cell with the corresponding NIR fluorescence signal. We included 29 EMR specimens in this analysis ( Table1 ).

The mean fluorescence intensity (MFI) values of areas at similar pathological stages were calculated for the multiple EMR specimens collected from individual patients (representative examples are shown in Fig. 1 c, d ). A pooled analysis of MFI was performed for nondysplastic versus dysplastic BE (neoplastic), within each individual EMR specimen and for every patient ( Fig. 1 a ). Areas containing either dysplastic or cancer tissue (neoplastic, Fig. 1 a ) had a significantly lower MFI ( *P* < 0.001) in comparison with areas that showed no dysplasia ( Fig. 1 a ) within the same EMR specimen ( Table 1 ).

### Correlation of fluorescence contrast with the extent of dysplasia in EMR specimen

In order to assess the sensitivity of the method for dysplasia detection, we analyzed the relationship between the spatial extent of dysplasia and the corresponding NIR fluorescence signal obtained for the same EMR specimen following topical application of WGA-IR800 ( Table 1 ).

Negative contrast (%) between areas of dysplasia and nondysplasia was defined as the ratio ( *D* – *ND* )/ *ND* × 100, where *D* and *ND* are the average MFIs for grid elements containing dysplastic and nondysplastic regions, respectively. This ratio reflects the contrast in fluorescence signal between dysplastic and nondysplastic areas within an EMR specimen, and corrects for differences in absolute fluorescence signal intensities that arose from inconsistent spraying of the EMR specimens on different days. By definition it could only be calculated for EMRs containing dysplasia.

An inverse linear correlation ( Fig. 1 b , Pearson *r* = −0.68, slope significantly non-zero, *P* = 0.002) was found between the negative contrast associated with dysplastic vs. nondysplastic regions within an EMR specimen and the corresponding spatial extent of dysplasia. Representative specimens are shown, containing low (20.5 %, Fig. 1 c ) and high (80.5 %, Fig. 1 d ) spatial extents of dysplasia, respectively.

### Fluorescence signal analysis of punch biopsies

In order to mimic the in vivo setting, we collected punch biopsies from areas within EMR specimens that showed differential fluorescence signals. We then calculated the MFI for locations targeted by the punch biopsies and investigated the relationship between the NIR fluorescence signal and the pathology grade for each individual biopsy. Fig. 2 a, b shows a representative EMR and corresponding biopsies, collected from dysplastic regions (I, high grade dysplasia [HGD], MFI = 0.091 ± 0.035; II, intramucosal cancer [IMC], MFI = 0.1539 ± 0.054) and a control nondysplastic region (III, MFI = 0.220 ± 0.008).

**Figure:**
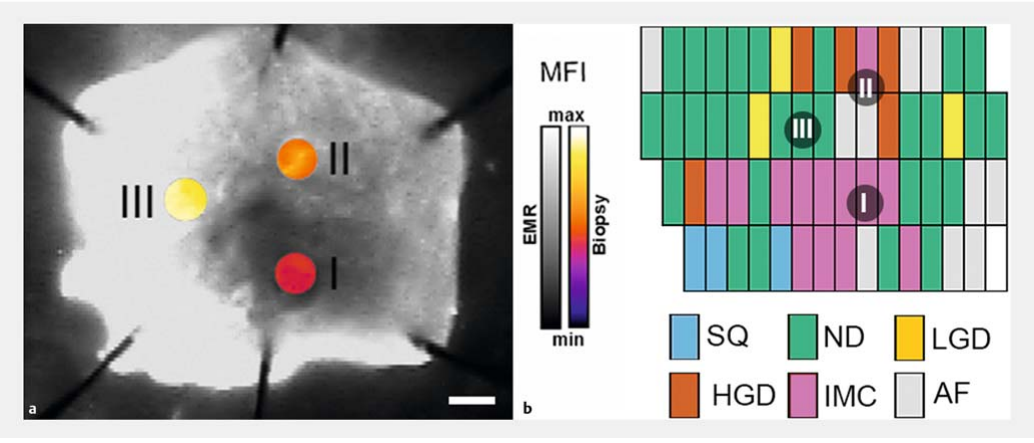


**Figure:**
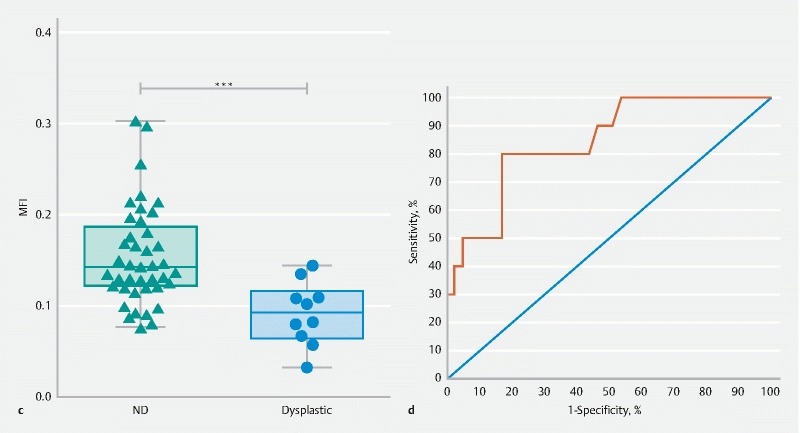


IMC can normally be detected based on standard high definition white light endoscopy. For this reason, and because the main aim of the technique is to improve detection of early dysplastic lesions (namely LGD or HGD), we excluded biopsies containing neoplasia (IMC). In a pooled analysis ( Fig. 2c ), biopsies collected from dysplastic regions that did not contain cancer, had significantly lower MFI ( *P* < 0.001) than biopsies collected from regions with nondysplastic Barrett’s epithelium. A receiver operating characteristic (ROC) analysis ( Fig. 2 d ), with a threshold of MFI = 0.1115, indicated an area under the curve (AUC) of 0.84 ± 0.07 ( *P* < 0.001), with a sensitivity of 80 % and a specificity of 82.9 %.

Biopsies collected from regions that did contain cancer, also had a significantly lower MFI ( *P* < 0.001) than biopsies collected from regions with nondysplastic BE. ROC analysis indicated an AUC, sensitivity, and specificity of 0.79 ± 0.06 ( *P* < 0.001), 75.0 % and 70.7 % respectively.

## Discussion

Three main classes of imaging agents, i. e. peptides 15 , antibodies 16 , and lectins 7 17 , delivered via topical or systemic routes, have been proposed for the optical detection of early neoplasia in the GI tract. Here, using WGA-IR800 as an imaging agent, applied topically to EMR specimens ex vivo, we observed a significant reduction of NIR fluorescence in areas containing dysplasia, in comparison with nondysplastic areas ( *P* < 0.001, Fig. 1a ). Higher negative contrast was obtained for EMRs containing larger areas of dysplasia and the spatial extent of the latter was found to correlate with NIR fluorescence imaging contrast ( Fig.1b ). A significant reduction of the NIR fluorescence signal (41.0 ± 13.6 %, *P* < 0.001) between nondysplastic and dysplastic tissue was also confirmed for a large cohort of punch biopsies ( Fig. 2c, n= 51) collected from EMR specimens, which was consistent with our previous studies 7 .

This study has three limitations. Firstly, our data are based on ex vivo experiments on EMR specimens using a commercially available wide-field fluorescence imaging system. Translation to the clinic will require the development of a NIR-capable endoscope, which is currently not available commercially. To address this limitation, we have recently developed a clinically applicable prototype bimodal NIR endoscope in preparation for this next step 18 . Secondly, most of the EMR specimens analyzed were collected from patients with advanced disease (either HGD or IMC), for which the differential in fluorescence intensity between dysplastic and nondysplastic tissue may be higher than that observed in patients with earlier stages of the disease. Thirdly, co-registration of the pathology grid with the planar fluorescence image is subject to error. To reach the best degree of approximation we have meticulously generated the pathology grid with readouts at every millimeter. We have also analyzed the extent of misregistration introduced by small shifts ( < 1 mm) of the pathology grid on the localized fluorescence signal and have found that the analysis conducted represents a worst-case scenario (data not shown). Moreover, analysis of the biopsy data ( Fig. 2 ) confirmed the results obtained from the whole EMR specimen ( Fig. 1 ). Future work to improve visualization could use alternative fluorescent dyes with more intense emissions (e. g. far-red, 650 – 750 nm).

In conclusion, our data suggest that molecular imaging with fluorescent lectins labeled with NIR dyes is a promising method for detecting early neoplasia in patients with Barrett’s esophagus. The next stage will be to conduct a first-in-human trial to evaluate the method in vivo. If lectin-based fluorescence endoscopy satisfied the minimum Preservation and Incorporation of Valuable Endoscopic Innovations (PIVI) criteria for detection of early neoplasia in Barrett’s esophagus 19 , the next step would be to apply this method to larger unselected surveillance populations.

## References

[1] Thrift A P Whiteman D C The incidence of esophageal adenocarcinoma continues to rise: analysis of period and birth cohort effects on recent trends Ann Oncol 2012 23 3155 3162 22847812 10.1093/annonc/mds181

[2] Fitzgerald R C di Pietro M Ragunath K British Society of Gastroenterology guidelines on the diagnosis and management of Barrett’s oesophagus Gut 2014 63 7 42 24165758 10.1136/gutjnl-2013-305372

[3] El-Serag H B Naik A D Duan Z Surveillance endoscopy is associated with improved outcomes of oesophageal adenocarcinoma detected in patients with Barrett’s oesophagus Gut 2016 65 1252 1260 26311716 10.1136/gutjnl-2014-308865

[4] Abrams J A Kapel R C Lindberg G M Adherence to biopsy guidelines for Barrett’s esophagus surveillance in the community setting in the United States Clin Gastroenterol Hepatol 2009 7 736 742; quiz 710 19268726 10.1016/j.cgh.2008.12.027 PMC3139243

[5] Boerwinkel D F Swager A Curvers W L The clinical consequences of advanced imaging techniques in Barrett’s esophagus Gastroenterology 2014 146 622 6.29E6 24412487 10.1053/j.gastro.2014.01.007

[6] Atreya R Goetz M Molecular imaging in gastroenterology Nat Rev Gastroenterol Hepatol 2013 10 704 712 23856892 10.1038/nrgastro.2013.125

[7] Bird-Lieberman E L Neves A A Lao-Sirieix P Molecular imaging using fluorescent lectins permits rapid endoscopic identification of dysplasia in Barrett’s esophagus Nat Med 2012 18 315 321 22245781 10.1038/nm.2616

[8] Liu Z Miller S J Joshi B P In vivo targeting of colonic dysplasia on fluorescence endoscopy with near-infrared octapeptide Gut 2013 62 395 403 22427239 10.1136/gutjnl-2011-301913 PMC3563943

[9] Tjalma J J Garcia-Allende P B Hartmans E Molecular fluorescence endoscopy targeting vascular endothelial growth factor A for improved colorectal polyp detection J Nucl Med 2016 57 480 485 26678613 10.2967/jnumed.115.166975

[10] ClinicalTrials.gov Bethesda (Maryland): National Library of Medicine (US). 200 Feb 29. Identifier CT02129933, VEGF-targeted[Internet]fluorescence near-infrared (NIR) endoscopy in (pre)malignant esophageal lesions (VICE) 24 4 2014Available from:https://clinicaltrials.gov/ct2/show/NCT02129933

[11] Sato K Nagaya T Choyke P L Near infrared photoimmunotherapy in the treatment of pleural disseminated NSCLC: preclinical experience Theranostics 2015 5 698 709 25897335 10.7150/thno.11559 PMC4402494

[12] Alam I S Neves A A Witney T H Comparison of the C2A domain of synaptotagmin-I and annexin-V as probes for detecting cell death Bioconjug Chem 2010 21 884 891 20402461 10.1021/bc9004415

[13] di Pietro M Boerwinkel D F Shariff M K The combination of autofluorescence endoscopy and molecular biomarkers is a novel diagnostic tool for dysplasia in Barrett’s oesophagus Gut 2015 64 49 56 24721904 10.1136/gutjnl-2013-305975 PMC4283667

[14] Dixon M F Gastrointestinal epithelial neoplasia: Vienna revisited Gut 2002 51 130 131 12077106 10.1136/gut.51.1.130 PMC1773259

[15] Burggraaf J Kamerling I M Gordon P B Detection of colorectal polyps in humans using an intravenously administered fluorescent peptide targeted against c-Met Nat Med 2015 21 955 961 26168295 10.1038/nm.3641

[16] Foersch S Kiesslich R Waldner M J Molecular imaging of VEGF in gastrointestinal cancer in vivo using confocal laser endomicroscopy Gut 2010 59 1046 1055 20639250 10.1136/gut.2009.202986

[17] Kuo J C Ibrahim A E Dawson S Detection of colorectal dysplasia using fluorescently labelled lectins Sci Rep 2016 6 24231 27071814 10.1038/srep24231 PMC4829854

[18] Waterhouse D J Joseph J Neves A A Design and validation of a near-infrared fluorescence endoscope for detection of early esophageal malignancy J Biomed Opt 2016 21 84001 27490221 10.1117/1.JBO.21.8.084001

[19] Sharma P Savides T J Canto M I The American Society for Gastrointestinal Endoscopy PIVI (Preservation and Incorporation of Valuable Endoscopic Innovations) on imaging in Barrett’s esophagus Gastrointest Endosc 2012 76 252 22817781 10.1016/j.gie.2012.05.007

